# Draft genome sequence of *Vreelandella stevensii* strain BS235 isolated from hypersaline lakes from Brazilian pantanal

**DOI:** 10.1128/mra.00748-24

**Published:** 2025-03-13

**Authors:** William Lautert-Dutra, Francine Melise dos Santos, Amanda Pasinato Napp, Clarissa Lovato Melo

**Affiliations:** 1 Environmental Monitoring and Biotechnology Laboratory, Institute of Petroleum and Natural Resources (IPR), Pontifical Catholic University of Rio Grande do Sul (PUCRS), Porto Alegre, Rio Grande do Sul, Brazil; Indiana University Bloomington, Bloomington, Indiana, USA

**Keywords:** carbon dioxide utilization, microbial metabolism, CO_2_ capture

## Abstract

Mitigating climate crisis has driven biotechnology research toward capturing and utilizing CO_2_. Production of biosurfactants by microorganisms’ metabolism offers a promising solution. We present the draft genome of *Vreelandella stevensii* strain BS235 from a hypersaline alkaline lake (Pantanal biome, Brazil). We identified genes related to CO_2_ metabolism and biosurfactant production.

## ANNOUNCEMENT

Producing alternatives to synthetic surfactants has driven research toward biotechnologies for biosurfactant production (1). Using carbon dioxide (CO_2_) as a carbon source for microorganisms presents a promising method for capturing and utilizing CO_2_ (2, 3). Several microorganisms produce biosurfactants, which reduce surface and interfacial tension biphasic, aiding processes like emulsification, dispersion, and interface stabilization (4–9). Thus, studying biosurfactant classes produced by environmental microorganisms is crucial. At the Institute of Petroleum and Natural Resources (IPR), we selected strain BS235 to explore the biotechnological capabilities related to CO_2_ in its metabolism and biosurfactant production.

Strain BS235 was isolated from hypersaline alkaline lake sediment in the *Pantanal Nhecolândia* region, Brazil (19°30′49″ S, 56°10′01.8″ W). The top layer of sediment was removed, and the apical portion was placed in 50 mL conical tubes at 4°C. Microorganisms were isolated through cultivation in saline medium (10 g/L yeast extract, 100 g/L NaCl, 3 g/L C_6_H_5_Na_3_O_7_, 2 g/L KCl, 1 g/L MgSO_4_, 280 µg/L MnCl_2_, 0.05 g/L FeSO_4_, and 3 g/L NaHCO₃) (10). The colonies underwent multiple successive subcultures to acquire pure cultures and final culturing in a nutrient-rich medium brain heart infusion (BHI) to detect potential contamination. Each isolate was verified using Gram staining to confirm bacterial cell morphology and ensure no contamination or mixed populations were present. Isolates were preserved in a culture collection at the LMA laboratory at IPR.

Microorganisms were lysed with lysis buffer (NeoSampleX). DNA extraction was manually performed using the magnetic beads technique and subsequently sequenced using Illumina NextSeq 1000 technology (2 × 300 bp, P1-600 Illumina Kit) at Neoprospecta Microbiome Technologies, Brazil. The quality of the 6,588,879 paired-end (PE) reads was analyzed using *FastQC* v0.11.9 (11), with low-quality reads removed and trimmed using *fastp* v0.23.4 (*trim-poly-g-detect-adapter minlenght* 90 bp). The total number of PE reads after trimming was 4,953,048 reads and fragment sizes with average lengths of 191 bp. Filtered reads were used in *SPAdes* v3.15.5 for *de novo* genome assembly (12). Coverage was estimated by mapping trimmed reads back to the assembly genome using *Bowtie2* v2.5.2 (13). The assembled quality was evaluated using *QUAST* v5.2.0 and *BUSCO* v5.5.6 (*bacteria_odb10*) (14, 15).

The final assembly showed 52 contigs with an estimated genome size of 3,840,077 bp (GC% content: 60.19; average coverage: 329×; N50: 312,925; L50: 5; BUSCO %C: 99.2). The scaffold was annotated using NCBI Prokaryotic Genome Annotation Pipeline (*PGAP)* build7061 (16) with the “-*taxcheck*” option to calculate the average nucleotide identity (ANI) for further taxonomic annotation (Table 1). The isolated BS235 showed high similarity with *Halomonas stevensii* S18214 (ANI = 97.49%).

**TABLE 1:** Annotation using the NCBI PGAP for *Vreelandella stevensii* strain BS235^*a*^

PGAP features	
Organism (input)	*Halomonas hydrothermalis*
Organism (output)	*Halomonas stevensii (ANI = 97.49%)*
Genes (total)	368
CDSs (total)	3,572
Genes (coding)	3,553
CDSs (with protein)	3,553
Genes (RNA)	108
rRNAs	7, 32, 5 (5S, 16S, 23S)
Complete rRNAs	3, 1 (5S, 23S)
Complete rRNAs	4, 32, 4 (5S, 16S, 23S)
tRNAs	60
ncRNAs	4
Pseudogenes (total)	19
CDSs (without protein)	19
Pseudogenes (ambiguous residues)	0 of 19
Pseudogenes (frameshifted)	2 of 19
Pseudogenes (incomplete)	17 of 19
Pseudogenes (internal stop)	2 out of 19
Pseudogenes (multiple problems)	1 of 19
CRISPR arrays	1

^*a*^ Coding sequences, CDS; Ribossomal RNA, rRNA; transfer RNA, tRNA; noncoding, ncRNA.

**Fig 1:**
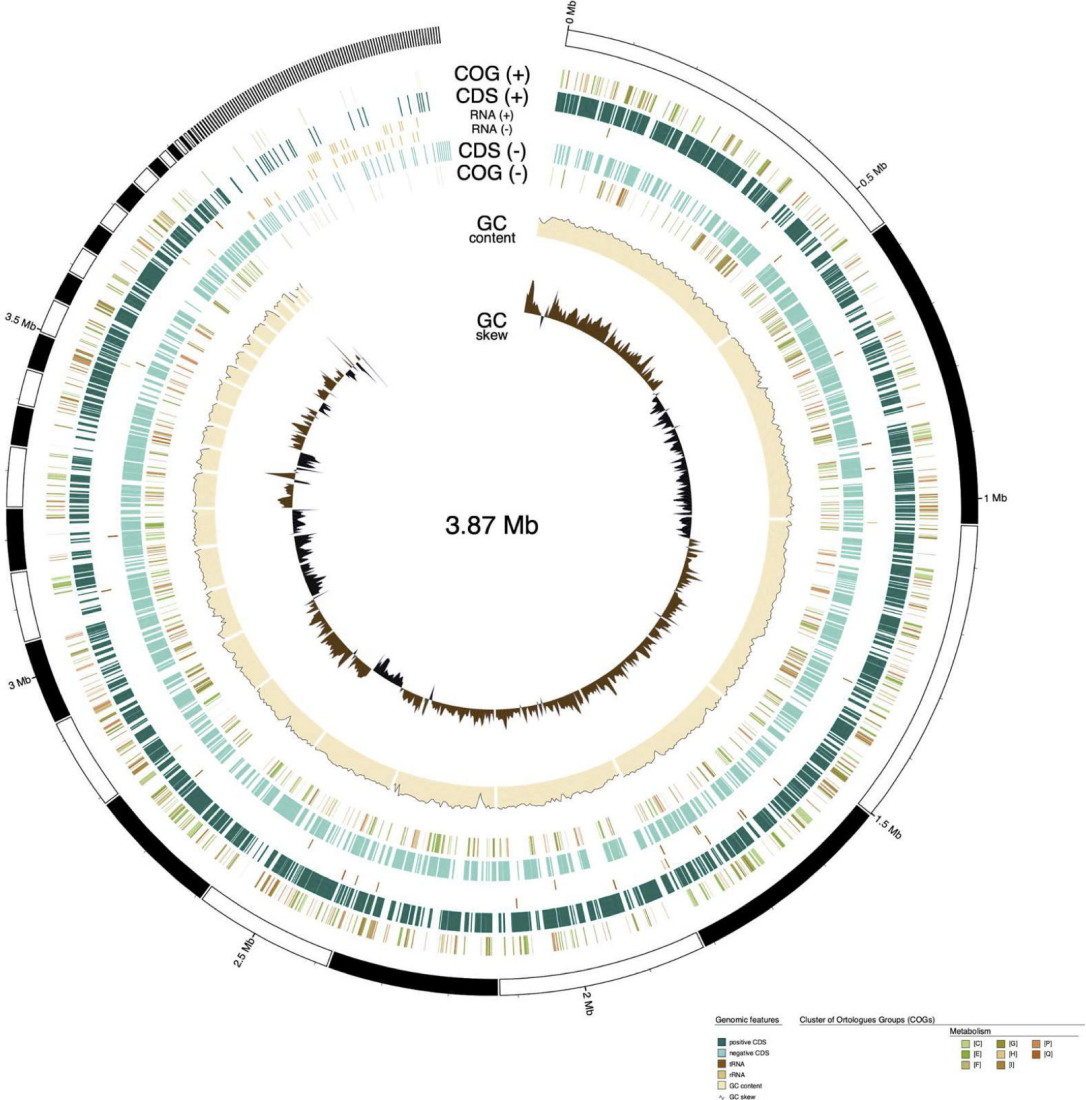
Overview of the genome of isolate 235. Circular representation of the complete genome of *Vreelandella stevensii* strain BS235. COG functional categories are displayed for the metabolism coding region predicted in the assembled genome. E, amino acid transport and metabolism; F, nucleotide transport and metabolism; G, carbohydrate transport and metabolism; H, coenzyme transport and metabolism; I, lipid transport and metabolism; P, inorganic ion transport and metabolism; Q, secondary metabolite biosynthesis, transport, and metabolism; R, general function prediction only; S, function unknown. Generated by GenoVi (https://github.com/robotoD/GenoVi).

Numerous genes related to biosurfactant production pathways and CO_2_ metabolism were identified in sequenced strains (*arfB*, *rhlA*, *rhlB*, *rpoN*, and *can*). The coding regions predicted in the annotation step were represented by functional categories called Cluster of Orthologous Group (COG) by the *GenoVi* v0.4.3 (17). The strain BS235 presented 76 genes classified as secondary metabolite biosynthesis, transport, and catabolism (Fig. 1).

The presence of these genes and COG categories highlights the potential of the 253 isolates for applications in CO_2_ capture and conversion into bioproducts like biosurfactants.

## Data Availability

The raw reads of this project and draft genome sequence are available under BioProject PRJNA1131744.
